# Jackstone in the Kidney: An Unusual Calculus

**DOI:** 10.1155/2021/8816547

**Published:** 2021-01-16

**Authors:** Serozsha Goonewardena, Umesh Jayarajah, Sanka Nalinda Kuruppu, Manoj Hilary Fernando

**Affiliations:** Department of Urology, National Hospital of Sri Lanka, Colombo, Sri Lanka

## Abstract

Jackstones are stones in the urinary tract that have the characteristic appearance resembling six-pointed toy jacks. They are nearly always reported to occur in the urinary bladder, and the occurrence in less capacious renal pelvis is unusual. We report a solitary, typical jackstone in the renal pelvis without significant outflow obstruction that was successfully treated with retrograde intrarenal surgery followed by extracorporeal shockwave lithotripsy. This highlights the complex pathophysiological mechanisms in stone formation which needs to be further studied. It is important to recognize the characteristic shape of the renal calculi on the radiological investigation in the diagnosis of the jackstones.

## 1. Introduction

Jackstones are stones in the urinary tract that have the characteristic appearance resembling six-pointed toy jacks. They are nearly always reported to occur in the urinary bladder [1, 2]. Interestingly, the first probable case of jackstone in the kidney was reported in 1906. Fowler in his paper described a similar stone and stated that “They occur in the renal pelvis, cysts of the kidney, or, as freely movable calculi, in the bladder” [3]. Fowler used words almost identical to those of Ord and Shattock way back in 1895 [4]. However, there is very limited literature about this rare type of calculus. Therefore, we report an unusual case of a jackstone in the kidney.

## 2. Case Presentation

**Figure 1:**
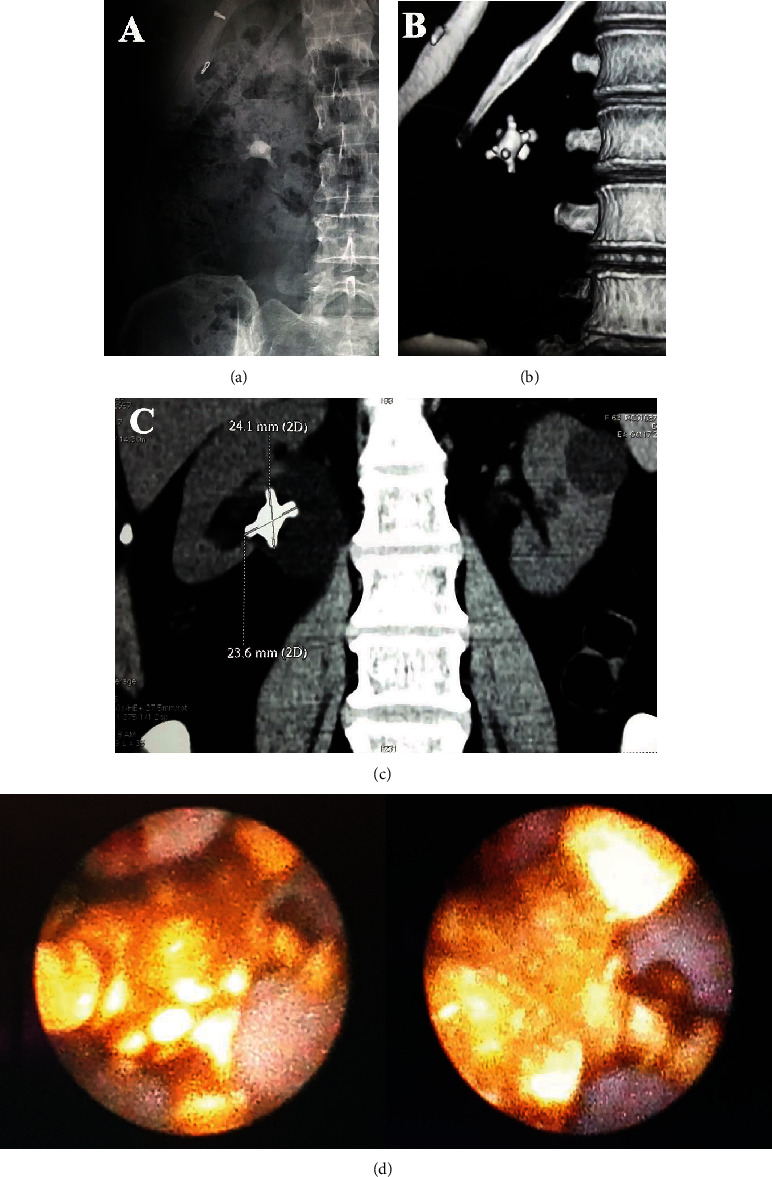
(a) X-ray: typical jackstone in the right kidney. (b) Reconstructed image of noncontrast computerized tomography (NCCT) confirming the presence of jackstone. (c) NCCT showing the right renal jackstone with mild hydronephrosis and no proximal hydroureter. (d) Endoscopic images showing a jackstone.

A 63-year-old woman presented with painless intermittent visible haematuria of 4 months duration with no history of flank pain, fever, or vomiting. She had type 2 diabetes mellitus for 18 years. Clinical examination was unremarkable. X-ray KUB showed a typical jackstone in the right kidney (Figure 1(a)). Noncontrast computerized tomography KUB (NCCT-KUB) confirmed the presence of jackstone in the renal pelvis of the right kidney with mild hydronephrosis and no proximal hydroureter (Figures 1(b) and 1(c)). The stone size was 2.4 cm × 2.3 cm, and the stone density was 1355 Hounsfield units. A 99 m Technetium-DTPA (Diethylenetriamine Penta-acetic Acid) diuretic renography ruled out significant obstruction with a differential function of 45% in the right kidney. She underwent cystoscopy which excluded bladder pathology accounting for visible haematuria. Right retrograde intrarenal surgery using a 7.5 Fr semirigid ureteroscope and Holmium : YAG lasertripsy completely fragmented the jackstone in the kidney (Figure 1(d)). A retrograde JJ stent was placed, and subsequent extracorporeal shockwave lithotripsy rendered her stone-free before the stent was removed. She had an unremarkable recovery. Facilities were not available for stone analysis.

## 3. Discussion

Jackstone in the kidney is exceedingly rare, and our review of the literature revealed only few previous reports [3, 5]. Grases et al. reported a 33-year-old man with two stones in the kidney of which the smaller stone (15 mm) was a jackstone. It had a nucleus of organic matter on which calcium oxalate monohydrate (COM) crystals have grown in a columnar concentric structure. The presence of organic matter in the urine acting as a nidus was postulated as a possible reason for the formation of the jackstone found in this patient. However, jackstones in the bladder may be composed of COM or calcium oxalate dihydrate (COD). COM (whewhellite) stones are usually smooth, whereas those composed of COD (Weddellite) tend to be irregular and yellow [2, 6].

Our case is unique as the patient presented with a solitary large jackstone (2.3 × 2.4 cm) that had a typical appearance as that of jackstones found in the bladder. In the previous case report by Grases et al., the typical appearance as seen with bladder jackstones was absent. The possible reason for the typical appearance in our patient may be the capacious renal pelvis that may have facilitated the formation of the jackstone. The composition of the jackstone in our patient is most likely COM. This is because the stone density was rather high (1355 HU), and its surface was relatively smooth (Figure 1(d)). In such a jackstone, Rao et al. found that COM is the main constituent [6]. Unfortunately, facilities were not available for stone analysis.

The exact pathophysiology of development of jackstone is poorly understood. Outflow obstruction remains the most common cause of bladder calculi in adults which was also seen with jackstones in the bladder [2]. However, interestingly in our patient, there was no significant obstruction at the pelviureteric junction demonstrated in the renogram curve.

We report a solitary, large, typical jackstone in the renal pelvis without significant outflow obstruction. This highlights the complex pathophysiological mechanisms in stone formation within the urinary system which needs to be further studied. It is important to recognize the characteristic shape of the renal calculi on the radiological investigation in the diagnosis of the jackstones.

## Data Availability

All data generated or analysed during this study are included in this published article.

## Abbreviations

KUB: Kidney-ureter-bladder
NCCT: Noncontrast computed tomography
DTPA: Diethylenetriamine pentaacetic acid
COM: Calcium oxalate monohydrate
COD: Calcium oxalate dihydrate.
